# Application of medical imaging in ovarian cancer: a bibliometric analysis from 2000 to 2022

**DOI:** 10.3389/fonc.2023.1326297

**Published:** 2023-12-04

**Authors:** Yinping Leng, Shuhao Li, Jianghua Zhu, Xiwen Wang, Fengyuan Luo, Yu Wang, Lianggeng Gong

**Affiliations:** ^1^ Department of Radiology, the Second Affiliated Hospital of Nanchang University, Nanchang, China; ^2^ Clinical and Technical Support, Philips Healthcare, Shanghai, China

**Keywords:** ovarian cancer, medical imaging, bibliometric analysis, collaborative network, Citespace

## Abstract

**Background:**

Ovarian cancer (OC) is the most lethal tumor within the female reproductive system. Medical imaging plays a significant role in diagnosis and monitoring OC. This study aims to use bibliometric analysis to explore the current research hotspots and collaborative networks in the application of medical imaging in OC from 2000 to 2022.

**Methods:**

A systematica search for medical imaging in OC was conducted on the Web of Science Core Collection on August 9, 2023. All reviews and articles published from January 2000 to December 2022 were downloaded, and an analysis of countries, institutions, journals, keywords, and collaborative networks was perfomed using CiteSpace and VOSviewer.

**Results:**

A total of 5,958 publications were obtained, demonstrating a clear upward trend in annual publications over the study peroid. The USA led in productivity with 1,373 publications, and Harvard University emerged as the most prominent institution with 202 publications. Timmerman D was the most prolific contributor with 100 publications, and Gynecological Oncology led in the number of publications with 296. The top three keywords were “ovarian cancer” (1,256), “ultrasound” (725), and “diagnosis” (712). In addition, “pelvic masses” had the highest burst strength (25.5), followed by “magnetic resonance imaging (MRI)” (21.47). Recent emergent keywords such as “apoptosis”, “nanoparticles”, “features”, “accuracy”, and “human epididymal protein 4 (HE 4)” reflect research trends in this field and may become research hotspots in the future.

**Conclusion:**

This study provides a comprehensive summary of the key contributions of OC imaging to field’s development over the past 23 years. Presently, primary areas of OC imaging research include MRI, targeted therapy of OC, novel biomarker (HE 4), and artificial intelligence. These areas are expected to influence future research endeavors in this field.

## Introduction

Ovarian cancer (OC) is the most lethal tumor of the female reproductive system (1). The absence of early clinical manifestations and effective screening techniques contributes to approximately 70% of patients being diagnosed at an advanced stage (2). The primary therapeutic approach for OC involves a combination of cytoreductive surgery and adjuvant chemotherapy (3). Despite notable advancements in chemotherapy and targeted therapy for OC, the prognosis remains unfavorable, with a 5-year overall survival rate ranging from 40% to 45% (4). Medical imaging, such as computed tomography (CT), magnetic resonance imaging (MRI), and positron emission tomography (PET)/CT, has been extensively employed for preoperative diagnosis, staging, treatment guidance, and prognostic assessment of OC (5–7). The utilization of medical imaging holds promise in enhancing the survival rate of OC patients through early detection and intervention (8). Over the past few decades, there has been a notable surge in the volume of scholarly publications pertaining to OC imaging across diverse academic domains (9). However, in the extensive literature database, it is often challenging for researchers to obtain a comprehensive and updated overview of the research trends and hotspots in this field.

Mathematical and statistical techniques are employed in bibliometrics to analyze published research regarding a specific subject area (10). This methodology allows not only the evaluation of research quality but also the identification of developing research trends and the prediction of possible future research directions (11, 12). Previous studies have utilized bibliometric analysis to examine prominent research topics and studies concerning OC (13–15). Duan’s research revealed that platinum-resistant OC is primarily focused on identifying populations that can benefit from immunotherapy alongside the practical implementation of immune checkpoint inhibitors (13). Baghban’s findings indicated an increasing trend in the research of extracellular vesicles and epithelial ovarian cancer (14). Conducting bibliometric analysis of literature pertaining to OC from various perspectives can aid in identifying research priorities within this domain while also presenting academic researchers with opportunities for collaboration (16, 17). However, the current research status, development trends, and future research directions of OC imaging are still unclear.

This study aims to use CiteSpace and VOSviewer for visual analysis, evaluate the current research status and development trends of OC imaging, and determine and summarize future research directions in this field.

## Materials and methods

### Data sources and search strategy

**Figure 1:**
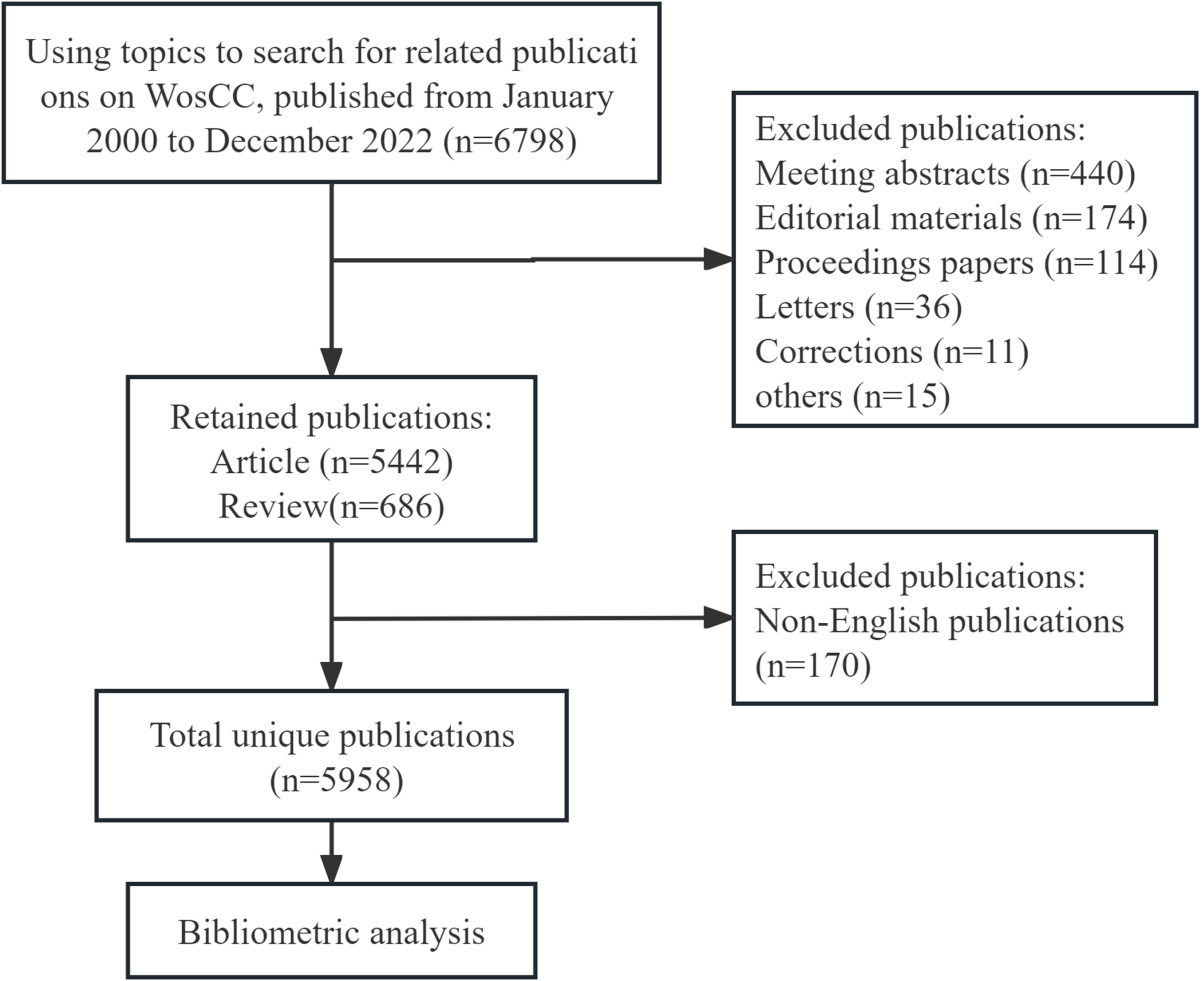
Flow chart of this study.

We conducted a literature search on August 9, 2023, to retrieve published literature from January 2000 to December 2022 from the Science Citation Index-Expanded (SCIE) of the Web of Science Core Collection (WoSCC). To avoid the bias of database updates, all searches were performed on the same day. The search strategy used was as follows: (TS = ((“ovarian neoplasm*”) OR (“ovarian cancer*”) OR (“ovarian carcinoma”) OR (“ovary cancer*”) OR (“ovary tumo*”) OR (“ovary neoplasm*”) OR (“ovary carcinoma”) OR (“ovarian tumo*”) OR (“cancer of ovary”) OR (“ovarian malignan*”) OR (“malignant ovarian*”)) AND TS = ((“CT”) OR (“computed tomography”) OR (“compute tomography”) OR (“magnetic resonance imaging”) OR (“MRI”) OR (“MR”) OR (“positron emission tomography”) OR (“PET”) OR (“single photon emission computed tomography”) OR (“SPECT”) OR (“ultrasonography”) OR (“ultrasound”))) AND FPY=(2000-2022). This study included only “articles” and “reviews” as publication types to ensure representativeness. Moreover, only documents written in English were considered. Two reviewers (YL and XW) independently screened the raw data extracting publications from WoSCC and eliminating duplicate/irrelevant documents. In cases of discrepancies, a third reviewer (LG) independently helped resolve them. The data were saved as a text file with Full Record and Cited References. A total of 5,958 publications were prepared for subsequent visual analysis. The study flowchart is presented in **Figure 1**. All data in this study were directly obtained from the database, and as no patients were involved, ethical declarations or approvals are not applicable.

### Data analysis and visualization

The bibliometric indicators included Price’s law, Lotka law, and Bradford’s law. Price’s law, a bibliometric production indicator, measures the productivity of a discipline or a country (18). Lotka uses the number of articles published to describe the frequency distribution of scientific productivity, often referred to as the “inverse square law of scientific production” (19). Bradford’s law, another bibliometric indicator, measures the dispersion of scientific information, illustrating the distribution of scientific literature within specific disciplines (20). Bradford proposed an information density-decreasing concentric zone productivity model.

The number of annual publications was imported into Excel 2019 and further analyzed to identify trends. CiteSpace (version 6.2.R4) was used to create knowledge maps of journals, institution co-authorship, references co-occurrences, and keyword co-occurrences. Time slices were set to 1 year per slice, and the g-index was selected as criteria for selection. The network pruning method was used for pruning. In the visual network maps, the size of nodes reflects the number of publications or the frequency of citations, while the connections between nodes indicate the strength of the connections. To detect emerging trends and sudden changes in research frontiers, the software’s “burst detection” and “betweenness centrality” functions were applied. Betweenness centrality is an index based on tree hole theory that measures the centrality of nodes in a network (21). Using CiteSpace with this index, the importance of relevant literature was measured, and purple circles indicated nodes with centrality greater than 0.1. The analysis of countries, co-cited countries, and co-cited institutions were performed using VOSviewer (version 1.6.19). Nodes and linear connections were present in the visual knowledge graph. Nodes in the graph represent key points, and node size represents the frequency of occurrence and citation.

## Results

This study examined 5,958 publications, including 5,299 articles (88.9%) and 659 reviews (11.1%). Our investigation showed that 26,643 authors from 3,958 institutions across 84 countries contributed to the production of 5,958 manuscripts in this study. These works were published in 1,186 journals, citing 126,115 references from 12,959 journals.

### Trends in publications

**Figure 2:**
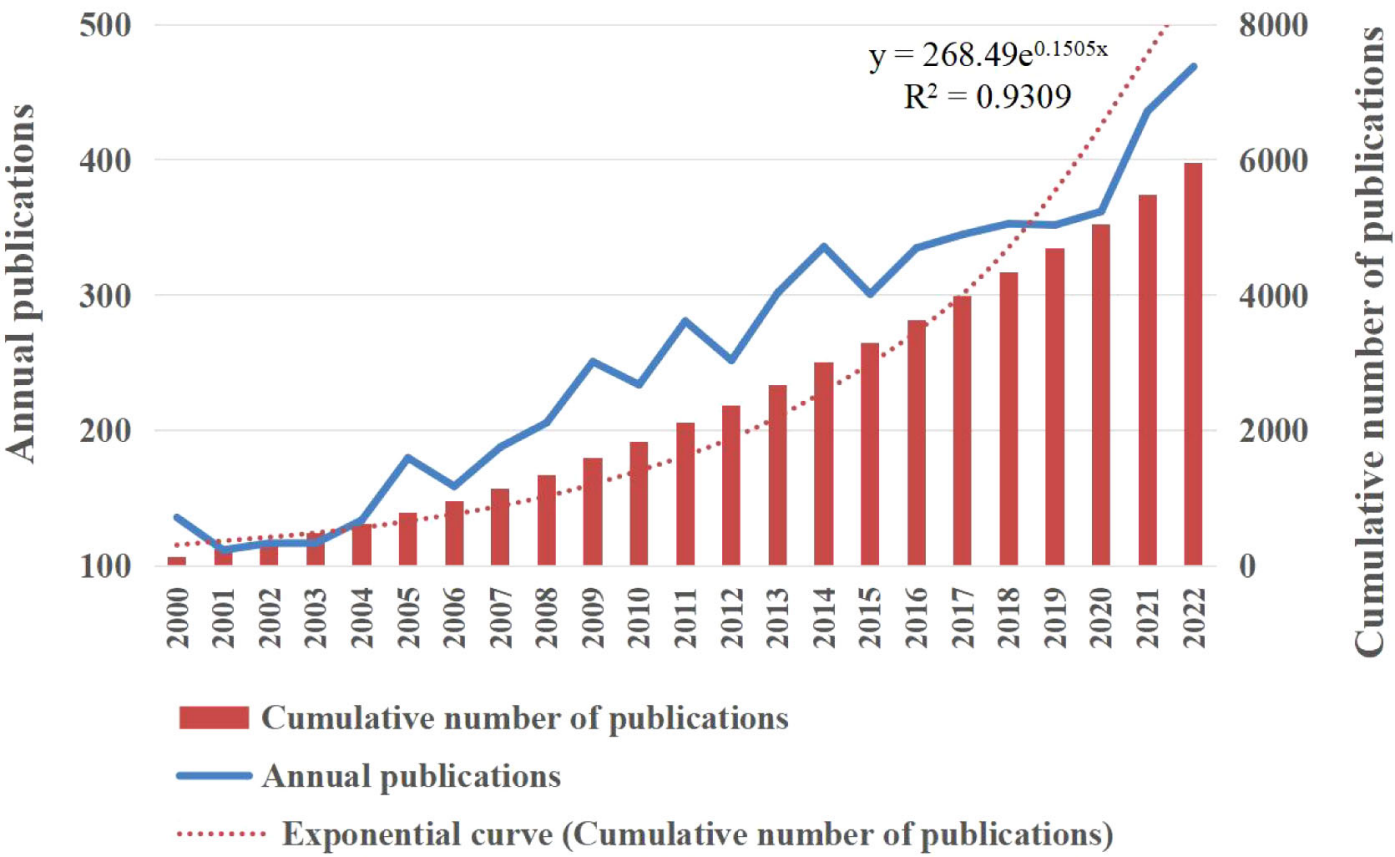
The number of publications and cumulative publications on ovarian cancer imaging from January 2000 to December 2022. Yearly publication count (y=268.49e^0.1505x^, r=0.970, where y is the cumulative publication number and x is the publication year).

To comprehend the evolution of related research, we examined the annual publication trends. The study period exhibited a discernible upward trajectory in annual publications (**Figure 2**). Moreover, the analysis indicated an average yearly production was around 259 studies, with 2022 recording the highest number of publications. **Figure 2** also illustrates the change in cumulative publications along the trend line, which followed the equation y = 268.49e^0.1505x^, with a correlation coefficient of 0.970, in accordance with Price’s law of exponential growth. The doubling time was determined to be 4.61 years, and the annual growth rate was 16.24%.

We used Lotka law to analyze the distribution of the authors and found that most of them were small producers, with a high transience index (occasional authors) of 76.5 (**Supplementary Table 1**). Bradford’s model was applied to assess the distribution of scientific journals publishing papers on OC imaging, as detailed in **Supplementary Table 2** delineating the material into Bradford’s zones.

### Analysis of countries and institutions

The publications identified came from 84 countries, with the USA leading in the number of studies (1,373 publications), constituting 23.04% of all documents. Subsequent top contributors included China (903 publications), Japan (417 publications), and Italy (402 publications) (**Table 1**). Among the 1,373 papers published in the USA, 1,116 were solely authored by individuals from the USA, while the remaining 257 papers involved collaborations with other countries. **Supplementary Figures 1A and B** display the cooperative relationships among countries and cited countries in OC imaging.

**Table 1:** Top 15 countries/regions in the ovarian cancer imaging research.

Rank	Country/ region	Counts	Percentage (%)	SCP	MCP	Cited country/ region	Citations	Average Citations
1	USA	1373	23.04	1116	257	USA	60347	44
2	China	903	15.16	824	79	United Kingdom	14857	42.8
3	Japan	417	7.00	402	15	China	11833	13.1
4	Italy	402	6.75	288	114	Italy	10181	25.3
5	United Kingdom	347	5.82	243	104	Germany	7888	43.3
6	Korea	213	3.58	199	14	Japan	7407	17.8
7	Germany	182	3.05	134	48	Netherlands	6326	44.5
8	France	168	2.82	119	49	Belgium	5613	56.1
9	Turkey	150	2.52	142	8	Canada	5196	41.6
10	Netherlands	142	2.38	96	46	France	4762	28.3
11	Canada	125	2.10	89	36	Korea	4295	20.2
12	Spain	118	1.98	71	47	Switzerland	3984	55.3
13	India	112	1.88	87	25	Australia	3847	37.7
14	Poland	103	1.73	83	20	Spain	2622	22.2
15	Australia	102	1.71	71	31	India	2324	20.8

SCP, the number of co-authored papers by authors of the same nationality; MCP, the number of co-authored papers with authors from others countries.

Research in OC imaging involved 3,958 institutions. The three institutions that had the most publications were Harvard University (USA, 202 publications), the University of London (United Kingdom, 190 publications), and the University of Texas System (USA, 168 publications) (**Table 2**). The institution with the highest centrality was the University of London (0.11), which indicates that this institution plays a crucial bridging role in research in this field. **Supplementary Figure 1C** shows the cooperative relationships among institutions in OC imaging.

**Table 2:** Top 15 institutions in the ovarian cancer imaging research.

Rank	Institution	Country	Count	Centrality	Year
1	Harvard University	USA	202	0.06	2000
2	University of London	United Kingdom	190	0.11	2000
3	University of Texas System	USA	168	0.02	2000
4	Catholic University of the Sacred Heart	Italy	138	0.01	2005
5	IRCCS Policlinico Gemelli	Italy	138	0.01	2005
6	KU Leuven	Belgium	135	0.08	2003
7	UTMD Anderson Cancer Center	USA	134	0.03	2000
8	Memorial Sloan Kettering Cancer Center	USA	133	0.07	2000
9	UDICE-French Research Universities	France	127	0.05	2004
10	University of California System	USA	112	0.09	2000
11	Harvard Medical School	USA	106	0.02	2000
12	Imperial College London	United Kingdom	102	0.06	2006
13	Fudan University	China	97	0	2012
14	National Institutes of Health	USA	95	0.07	2000
15	University College London	United Kingdom	95	0.02	2006

### Analysis of authors and co-cited authors

The total number of authors across 5,958 publications was 26,643, yielding a co-authorship index of 4.47. **Table 3** shows the top 15 authors and co-cited authors. Timmerman D led in publications (100), followed by Testa AC (83) and Valentin L (77). Timmerman D also emerged as the most co-cited author (2,650 citations, H_index: 40), followed by Valentin L (2,248 citations, H_index: 36) and Van Holsbeke C (1,547 citations, H_index: 31).

**Table 3:** Top 15 authors and co-cited authors in the ovarian cancer imaging research.

Rank	Author	Counts	H_index	Co-cited author	Citations	H_index
1	Timmerman D	100	40	Timmerman D	2650	40
2	Testa AC	83	30	Valentin L	2248	36
3	Valentin L	77	36	Van Holsbeke C	1547	31
4	Scambia G	60	21	Vergote I	1478	33
5	Alcazar JL	55	24	Testa AC	1466	30
6	Guerriero S	55	26	Bourne T	1425	30
7	Van Holsbeke C	55	31	Van Calster B	1354	29
8	Bourne T	51	30	Guerriero S	1004	26
9	Menon U	50	26	Van Huffel S	958	20
10	Vergote I	50	33	Jurkovic D	951	22
11	Sala E	48	23	Hricak H	828	19
12	Dyson PJ	47	26	Fischerova D	809	23
13	Fischerova D	45	23	Savelli L	793	21
14	Wang Y	39	13	Fruscio R	702	18
15	Van Calster B	38	29	Kaijser J	647	16

### Analysis of active journals

**Figure 3:**
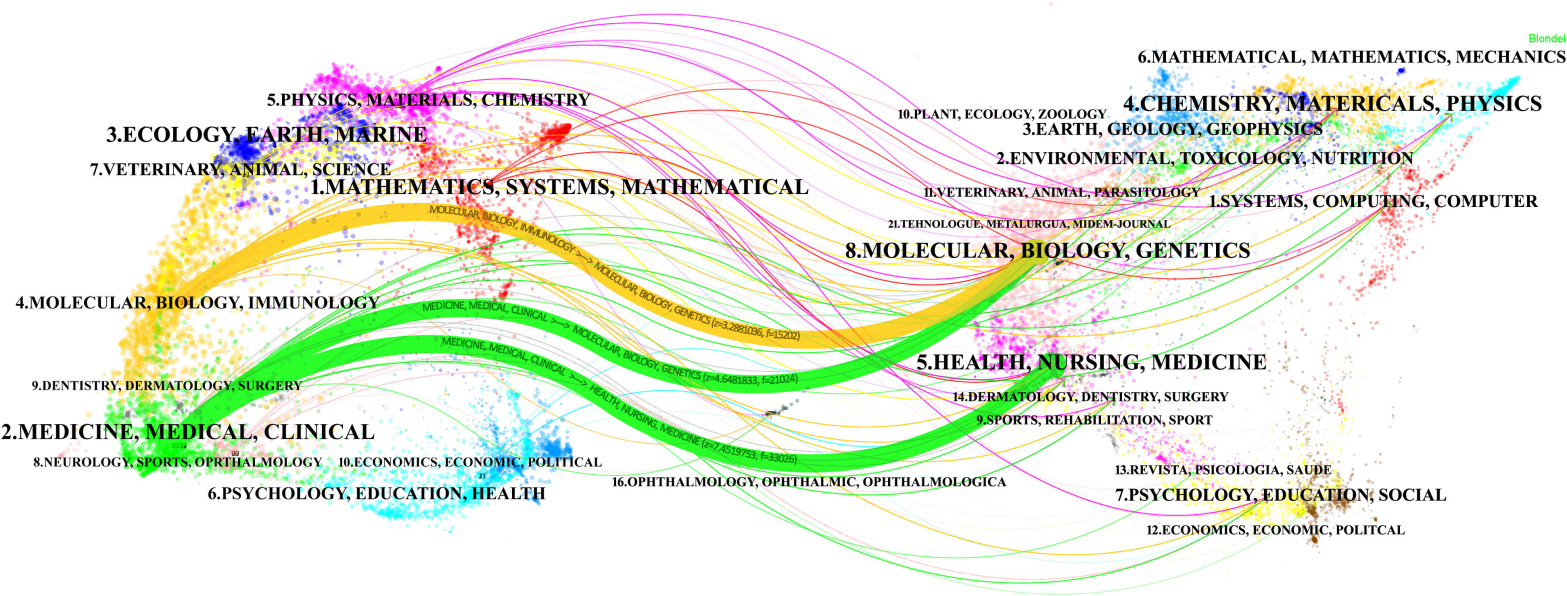
The dual-map overlay of journals related to ovarian cancer imaging research. The left side has the citing journals, and the right has the cited journals. The color of the path indicates the citation relationship. In the citing map, the length of the ellipse’s vertical axis reflects the number of papers published by the journal, and the length of the ellipse’s horizontal axis reflects the number of authors in the journal.

A total of 1,186 journals contributed to this topic. **Table 4** outlines the top 15 journals in OC imaging, with Gynecologic Oncology leading in publications [296 publications, Impact factor (IF): 5.3], trailed by the International Journal of Gynecological Cancer (182 publications, IF: 4.66). Journal of Clinical Oncology claimed the highest IF (50.72). The top 15 cited journals are shown in **Table 4**. Gynecologic Oncology also had the most citations (12,493 citations), followed by Journal of Clinical Oncology (6,104 citations) and Radiology (5,284 citations). **Figure 3** shows a dual-map overlay of the citing and cited journals in OC imaging research. The left labels were the citing journals, and the right labels were the cited journals. These labels represented the disciplines covered by the journals. There are three main citation paths, are highlighted in yellow and green. The yellow path indicated that studies from the molecular, biology, and genetics journals were cited in studies from the molecular, biology, and immunology journals. The two green paths indicated that studies from the molecular/biology/genetics and health/nursing/medicine journals were cited in studies from the medicine/medica/clinical journals.

**Table 4:** The top 15 journals and cited journals related to ovarian cancer imaging.

Rank	Journal	Counts	Citations	IF (2022)	Cited journals	Citations	Centrality
1	Gynecologic Oncology	296	12493	5.30	Gynecologic Oncology	12493	0.28
2	International Journal of Gynecological Cancer	182	4110	4.66	Journal of Clinical Oncology	6104	0.15
3	European Journal of Gynaecological Oncology	153	621	0.26	Radiology	5284	0.14
4	Ultrasound in Obstetrics & Gynecology	144	3843	8.68	International Journal of Gynecological Cancer	4110	0.03
5	European Radiology	66	2234	7.03	Obstetrics Gynecology	3474	0.01
6	Medicine	63	198	1.82	American Journal of Obstetrics and Gynecology	2796	0.03
7	Journal of Nuclear Medicine	61	2772	11.08	New England Journal of Medicine	2694	0.02
8	Journal of Clinical Oncology	60	6104	50.72	Cancer	2298	0.03
9	Journal of Ovarian Research	59	476	5.51	Cancer Research	1362	0.17
10	PLoS One	59	1083	3.75	American Journal of Roentgenology	1351	0.05
11	Archives of Gynecology and Obstetrics	58	576	2.49	British Journal of Cancer	1338	0.11
12	Journal of Obstetrics and Gynecology Research	58	300	1.69	Clinical Cancer Research	1245	0.11
13	Radiology	58	5284	29.15	CA-A Cancer Journal for Clinicians	1192	0.02
14	Anticancer Research	56	873	2.44	Lancet	1129	0.08
15	Frontiers in oncology	50	301	5.74	European Journal of Cancer	1107	0.04

IF, impact factor.

### Analysis of cited references

**Figure 4:**
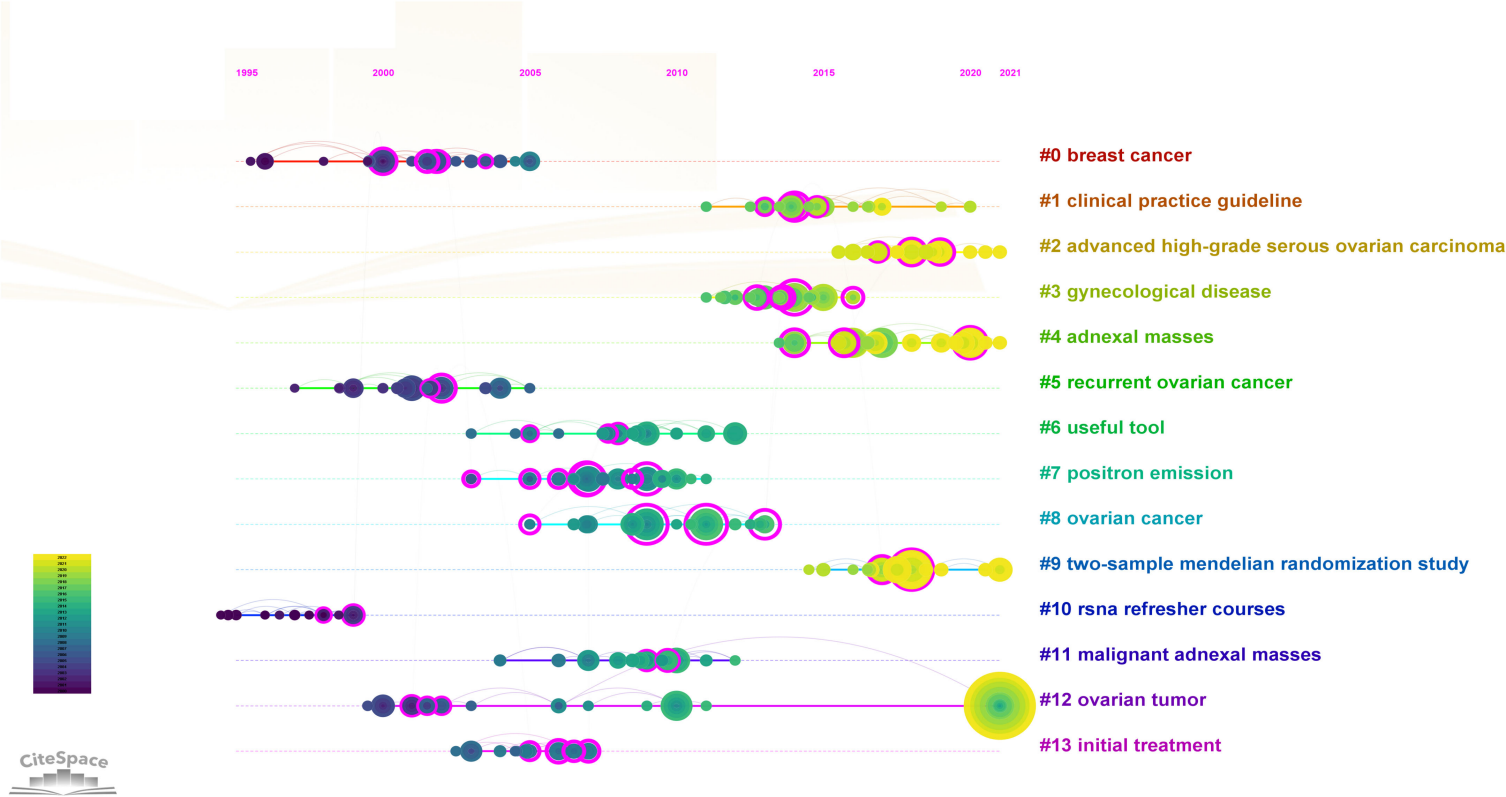
Timeline view of reference. In the timeline view, different colors of nodes on the same line indicate different years. Therefore, the nodes on the left represent older references, while the nodes on the right represent more recent references. A straight line in the same horizontal position indicates the set of all clustered references belonging, and the cluster label is located at the line’s rightmost end. The first cluster label on the knowledge map was “#0 breast cancer” and the second cluster label was “#1 clinical practice guideline”. Node size represents co-citation frequency, and the links between nodes indicate co-citation relationships. The occurrence year of each node indicates the initial co-citation time.

The references with the most citations are often considered foundational to research in a specific field. **Table 5** shows the top 15 co-cited references in OC imaging research. As mentioned above, the article “Terms, definitions and measurements to describe the sonographic features of adnexal tumors: a consensus opinion from the International Ovarian Tumor Analysis Group” by Timmerman D published in Ultrasound In Obstetrics & Gynecology, was the most cited reference (298 citations). **Figure 4** provides a timeline view of the co-cited references. The largest cluster was “#0 breast cancer”, followed by “#1 clinical practice guideline”, and “#2 advanced high-grade serous ovarian carcinoma”. The timeline shows that “#0 breast cancer” and “#10 rsna refresher courses” constituted the earliest clusters. Additionally, it is noteworthy that “#2 advanced high-grade serous ovarian carcinoma”, “#4 adnexal masses” and “#9 two-sample mendelian randomization study” became the most popular research topics in recent years. Five out of the 13 clusters are still active, suggesting that these research directions remain vibrant and represent ongoing hotspots in OC imaging.

**Table 5:** The top 15 co-cited references in the ovarian cancer imaging research.

Rank	Title	First author	Journal	Year	Citations
1	Terms, definitions and measurements to describe the sonographic features of adnexal tumors: a consensus opinion from the International Ovarian Tumor Analysis (IOTA) Group.	Timmerman D	Ultrasound In Obstetrics & Gynecology	2000	298
2	A risk of malignancy index incorporating CA 125, ultrasound and menopausal status for the accurate preoperative diagnosis of ovarian cancer.	Jacobs I	British Journal of Obstetrics and Gynaecology	1990	278
3	Cancer statistics, 2019.	Siegel RL	CA-A Cancer Journal For Clinicians	2021	275
4	Survival effect of maximal cytoreductive surgery for advanced ovarian carcinoma during the platinum era: a meta-analysis.	Bristow R E	Journal of Clinical Oncology	2002	194
5	Simple ultrasound-based rules for the diagnosis of ovarian cancer.	Timmerman D	Ultrasound In Obstetrics & Gynecology	2008	186
6	Neoadjuvant chemotherapy or primary surgery in stage IIIC or IV ovarian cancer.	Vergote I	New England Journal of Medicine	2010	171
7	Transvaginal sonographic characterization of ovarian disease: evaluation a new scoring system to predict ovarian malignancy.	Sassone A M	Obstetrics and gynecology	1991	148
8	Staging of advanced ovarian cancer: comparison of imaging modalities–report from the Radiological Diagnostic Oncology Group.	Tempany CM	Radiology	2000	144
9	Peritoneal Metastases: Detection with Spiral CT in Patients with Ovarian Cancer.	Fergus V	Radiology	2002	137
10	A model for predicting surgical outcome in patients with advanced ovarian carcinoma using computed tomography.	Bristow RE	Cancer	2000	126
11	Sensitivity and specificity of multimodal and ultrasound screening for ovarian cancer, and stage distribution of detected cancers: results of the prevalence screen of the UK Collaborative Trial of Ovarian Cancer Screening.	Menon U	Lancet Oncology	2009	125
12	Effect of screening on ovarian cancer mortality: the Prostate, Lung, Colorectal and Ovarian (PLCO) Cancer Screening Randomized Controlled Trial.	Buys SS	JAMA-Journal of the American Medical sociation	2011	124
13	Evaluation of a risk of malignancy index based on serum CA125, ultrasound findings and menopausal status in the pre-operative diagnosis of pelvic masses.	Tingulstad S	British Journal of Obstetrics and Gynaecology	1996	123
14	New response evaluation criteria in solid tumours: revised RECIST guideline (version 1.1).	Eisenhauer EA	European Journal of Cancer	2009	118
15	A novel multiple marker bioassay utilizing HE4 and CA125 for the prediction of ovarian cancer in patients with a pelvic mass.	Moore RG	Gynecologic Oncology	2009	117

### Analysis of keywords

**Figure 5:**
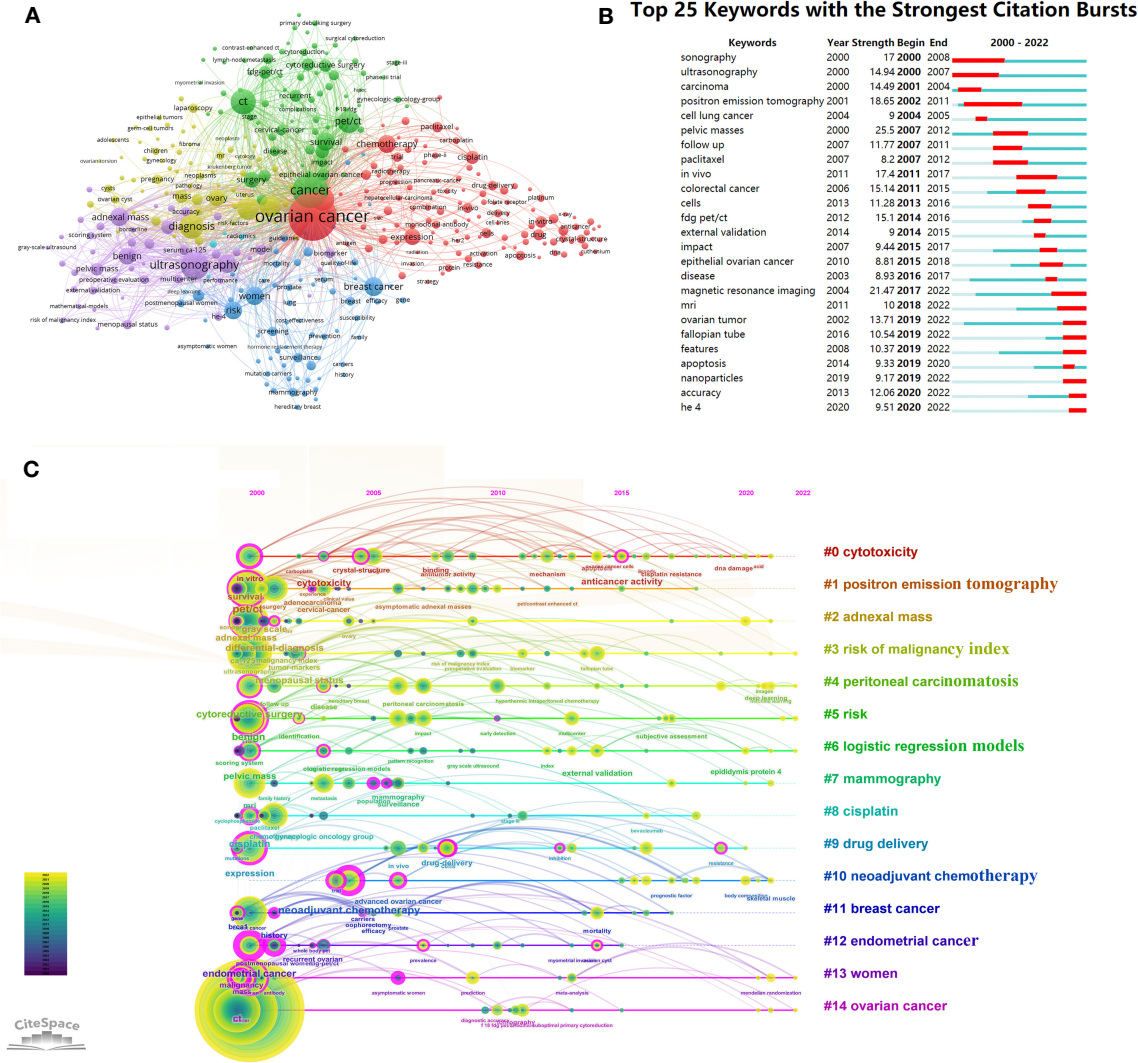
Analysis of ovarian cancer imaging-related keyword. **(A)** The co-occurrence network of keywords in VOSviewer. The figure displays the keywords that occurred more than 20 times. The nodes with different colors represent the keywords from different clusters, and the size of the nodes reflects their frequency. **(B)** The 20 keywords with the highest citation bursts in ovarian cancer imaging research. The red bold line indicates the years of citation bursts. **(C)** Timeline analysis of the keywords related to ovarian cancer imaging. The first cluster label on the knowledge map was “#0 cytotoxicity” and the second cluster label was “#1 positron emission tomography”. Each keyword cluster had its own timeline. Each node represents a keyword, which was marked under the node. The order in which nodes appear indicates the development and evolution process of keywords under clustering.

Keywords can summarize the main content of publications and exploring the frontiers of OC imaging research. To enhance the relevance to this study, the selection of keywords excluded “cancer” and “carcinoma”. The top 15 keywords in OC imaging research are shown in **Supplementary Table 3**. The keyword “ovarian cancer” (1,256), followed by “ultrasound” (725), “diagnosis” (712) with the highest frequency. **Figure 5A** shows a collaboration network of keywords created by VOSviewer. The research direction grouped the keywords and roughly divided them into 6 categories: the red cluster was the largest one, primarily focused on radiotherapy, chemotherapy, and anticancer treatment of OC. The green cluster was involved with the initial surgical evaluation of epithelial ovarian cancer and the prognostic evaluation of ovarian cancer with CT and PET/CT. The dark-blue cluster involved the risk factors of women suffering from OC and the diagnosis of OC related to breast cancer. The purple cluster was associated with the ovarian cancer risk of malignant index (RMI), and the ultrasound evaluation adnexal quality score. The yellow cluster was mainly related to the diagnosis and differential diagnosis of ovarian cancer. The baby-blue cluster was associated with the radiomics and deep learning research in ovarian cancer. **Figure 5B** displays the top 25 keywords with the highest burst strength in OC imaging research. “pelvic masses” had the highest burst strength (25.5), followed by “MRI” (21.47), which extends until 2022. “positron emission tomography” had the longest duration, from 2002 to 2011. Human epididymis protein 4 (HE 4) was a new keyword starting in 2020, with an burst strength of 9.51. Recently, popular keywords such as “apoptosis”, “nanoparticles”, “features”, “accuracy”, and “HE 4” reflect research trends in this field and may become research hotspots in the future.

Using the timeline viewer for keywords analysis reveals evolving hotspots in the field over time. The most common keywords for each group over time were shown by the timeline graph in CiteSpace (**Figure 5C**). The earliest and largest cluster was “#0 cytotoxicity”. Among the earliest keywords in this field were “carboplatin” and “crystal-structure”, while “DNA damage” and “acid” were the latest research targets in this area. The “#1 positron emission tomography” cluster was another large cluster that emerged earlier. Research frontiers in this field were keywords such as “anticancer activity” and “cisplatin resistance”. In addition, five of the 14 clusters are still active, including, “#3 risk of malignancy index”, “#4 peritoneal carcinomatosis”, “#5 risk”, “#6 logistic regression models”, “#13 women”, and “#14 ovarian cancer”, indicating that relevant research continues to progress in these specific areas.

## Discussion

### General information

In this study, we conducted a systematic and comprehensive bibliometric analysis of research on OC medical imaging from January 1, 2000 to December 31, 2022. The number of annual publications showed a clear upward trend for OC imaging studies, indicating the sustained and significant attention this field has received in recent years. This study provides an overview of the medical imaging applications of ovarian cancer, helping researchers in understanding the current status, collaborative networks, and primary research hotspots in this field. In addition, our research findings provide a series of suggestions for future investigation.

To promote teamwork and global collaboration in this area, we analyzed the distribution of countries/regions and institutions. Of the 5,958 publications, 23.04% were completed by researchers in the USA. China, as the only developing country in the top five productivity rankings, held the second position in OC imaging research output, following the USA. Moreover, seven of the 15 most productive institutions were in the USA. Harvard University, affiliated with the USA, having the most publications and the highest attention level. Therefore, the USA made a significant contribution to the academic influence and reputation of OC imaging research. While the University of London did not have the highest number of publications, it demonstrated the highest centrality, indicating its important bridging role in inter-country cooperation. Analyzing journals and co-cited journals can assist researchers in choosing suitable journals for their papers. The majority of papers were published in *Gynecological Oncology*, *International Journal of Gynecological Cancer*, and *European Journal of Gynecological Oncology*, all belonging to the gynecological and oncology fields. Meanwhile, *Gynecological Oncology* and *Journal of Clinical Oncology* stood out as the most cited journals.

The degree of correlation between studies can be measured by references cited by other publications together. Co-citation analysis can help researchers identify the common knowledge bases shared by multiple studies efficiently and conveniently (22, 23). Analyzing the most co-cited references, reveals that advanced high-grade serous ovarian cancer and adnexal masses are important research foundations for OC imaging. Belgian researcher Timmerman D played a pivotal role by first standardizing the terminology, definitions, and measurements for describing sonographic features of adnexal masses (24). Several scholars have investigated the combination of ultrasound and cancer antigen 125 (CA125) to evaluate the malignancy risk index of advanced OC, which has yielded positive outcomes (25, 26). Subsequently, researchers explored the treatment of advanced OC (27, 28). During the platinum era, maximal cytoreductive surgery emerged as one of the most important determinants of survival in stage III or IV OC patients (27). Among them, Vergote I’s article, which was published in the journal New England Journal of Medicine, reported that the survival time of interval debulking surgery following neoadjuvant chemotherapy was not inferior to that of chemotherapy after maximal cytoreductive surgery in patients with stage IIIC or IV OC (28).

### Current research status of imaging in OC

CT is the recommended imaging technique for staging OC. The overall accuracy of CT for diagnosis of malignant ovarian masses is reported to be as high as 89%. However, the sensitivity and specificity of CT in diagnosing malignant abdominal lymph nodes were 41% and 89% (29). A recent meta-analysis showed that CT’s sensitivity, specificity, and diagnostic advantage rate in detecting peritoneal metastasis were 68%, 88%, and 15.9%, respectively (30). But CT appears to be relatively accurate in predicting the involvement of the diaphragm and omentum. Additionally, CT has been employed to predict primary cytoreductive outcomes for advanced OC. Axtell et al. demonstrated that the sensitivity and specificity of CT prediction for optimal suboptimal surgical outcome in patients with advanced OC were 79% and 75%, respectively (31).

PET/CT has limitations in characterizing ovarian masses, but proves valuable for OC staging and detecting recurrent diseases. Due to limited spatial resolution, PET/CT may struggle to identify peritoneal tumor deposits smaller than 1 cm. Another indication for PET/CT is to detect recurrent diseases. A meta-analysis showed that compared with CT and MRI, PET/CT was relatively accurate in detecting recurrence, with sensitivity and specificity of 91% and 88%, respectively (32).

MRI provides excellent tissue differentiation and serve as a problem-solving tool to characterize uncertain lesions observed on CT or ultrasound. In a recent meta-analysis showed that the sensitivity and specificity of MRI in diagnosing OC were 91% and 85%, respectively, surpassing CT and PET/CT in OC detection (33). In a meta-analysis, CT, PET/CT, and MRI were compared for detecting peritoneal metastasis, and MRI showed the highest combined regional sensitivity and specificity, with 92% and 84%, respectively (30).

Ultrasound plays an important role in the initial evaluation of adnexal masses and high-risk patients screening. It is recommended to use a vaginal ultrasound morphological scoring system to distinguish between benign and malignant ovarian lesions. The sensitivity and specificity of this system were 100% and 83%, respectively (26). Although ultrasound can be used for diagnose OC, it is not the preferred imaging method for staging OC. In patients with advanced OC, the sensitivity of ultrasound in detecting peritoneal metastasis is relatively low (69%) (34).

### Research hotspots and frontiers

Keyword analysis provides valuable insights into the main research hotspots and directions in the field of OC imaging from 2000 to 2022. Ultrasound emerges as a pivotal focus in OC imaging research, particularly in the initial assessment of adnexal masses and screening individuals with a high risk of OC. RMI based on ultrasonography, menopausal status, and serum CA125 levels can quantitatively assess the risk of malignancy for accurate preoperative diagnosis of OC (25). Analyzing keyword bursts can help researchers in identifying the frontier or future trends in specific fields (35). The keyword with the highest burst intensity is pelvic masses, spanning from 2007 to 2012. During this period, research on pelvic masses mainly focused on evaluating the efficacy of paclitaxel in OC treatment and subsequent follow-up outcomes. Additionally, PET has historically been a prominent avenue in OC imaging with PET/CT proving advantageous in staging, prognostic prediction, response evaluation, and restaging of OC patients (36). MRI has emerged as a research hotspot in the field of OC imaging. MRI has high soft tissue resolution and can be used as a problem-solving tool to characterize uncertain lesions observed on CT or ultrasound (37). MRI not only reduces unnecessary surgical interventions for benign lesions but also minimizes surgeries in inoperable cases, facilitating the initiation of initial chemotherapy treatments for patients and enhancing their overall treatment experience.

In recent times, the research community has shown growing interest in keywords such as “HE 4”, “features”, “accuracy”, “apoptosis” and “nanoparticles”, which are indicative of emerging research trends in this particular field and have the potential to become significant areas of investigation in the future.

Notably, HE 4 has emerged as a novel biomarker for ovarian cancer. Mi’s research indicates that serum HE4 levels can contribute to diagnosing, evaluating treatment responses, and predicting recurrence in patients with advanced ovarian cancer, fallopian tube cancer, and peritoneal cancer (38). Zhao used CA125 and HE 4 to detect OC, found that the sensitivity of CA125 was higher than that of HE 4 (88.2% vs. 54.7%), while the specificity of HE 4 was higher than that of CA125 (97.9% vs. 67.4%) (39). A prospective multicenter study shown that the risk of ovarian malignancy algorithm based on the CA125 and HE 4 levels can be used to evaluate the risk of epithelial ovarian cancer in premenopausal and postmenopausal women with pelvic masses (40).

The term “apoptosis” and “nanoparticles” refers primarily to the targeted therapy associated with OC. In recent years, nanomedicines have been widely used in multiple therapeutic fields, especially in the field of cancer. The use of nanomedicines has greatly improved the safety and efficacy of common anticancer drugs. Nanodrugs have targeted and sustained-release properties. At present, liposomal doxorubicin has been applied in clinical treatment of recurrent platinum resistant OC patients (41). Nanoparticle albumin-bound paclitaxel demonstrated therapeutic efficacy in the treatment of OC, with minimal toxic side effects and improved patient acceptance and compliance (42).

The analysis of the keyword timeline, reveals that advancements in imaging and computer technology have significantly enhanced the precision of investigating OC. Furthermore, the evolution of computer science has propelled the growth of artificial intelligence (AI), with radiomics and deep learning emerging as a subfield of AI. Radiomics in OC relies on imaging data for predicting pathological diagnosis, recurrence risk, and treatment prognosis (43, 44). However, the current research on deep learning in OC is still in its preliminary stages (44, 45). Consequently, AI in the domain of OC imaging holds potential as a future development trend.

This study has some limitations. First, the search was limited to the WoSCC SCIE database due to software compatibility constraints, potentially resulting in the omission of relevant research findings. Second, the bibliometric analysis conducted using CiteSpace and VOSviewer focused primarily on principal conclusions rather than providing a comprehensive examination of the entire text, limiting the capacity for a systematic review. Third, it is important to note that CiteSpace software does not distinguish between the first author and the corresponding author. Additionally, the presence of multiple authorship identities for the same author may introduce biases in the results related to affiliated institutions.

## Conclusions

The number of publications in OC imaging is gradually increasing with the USA and China being the primary contributors to OC imaging research. Among the authors, Timmerman D from Belgium stands out as the most prolific and highly cited. Gynecologic Oncology is the journal with the highest number of publications and citations in this area. Currently, the main areas of focus in OC imaging research encompass MRI, targeted therapy for OC, novel biomarker (such as HE4), and AI. These areas are expected to shape future research endeavors in the field of OC imaging.

## Data availability statement

The raw data supporting the conclusions of this article will be made available by the authors, without undue reservation.

## Author contributions

YL: Data curation, Formal Analysis, Writing – original draft. SL: Conceptualization, Investigation, Writing – review & editing. JZ: Methodology, Software, Writing – original draft. XW: Data curation, Methodology, Writing – original draft. FL: Formal Analysis, Methodology, Software, Writing – original draft. YW: Investigation, Writing – review & editing. LG: Supervision, Writing – review & editing, Formal Analysis.
