# Supplementary material for: Application of medical imaging in ovarian cancer: a bibliometric analysis from 2000 to 2022
Source: Front Oncol. 2023 Dec 4;13:1326297. doi: 10.3389/fonc.2023.1326297 (PMC10725957; doi:10.3389/fonc.2023.1326297)
Supplement: Supplementary file 1 [file DataSheet_1.docx]

| Contents | Pages |
| --- | --- |
| Supplementary Tables (1, 2, 3) | Pages 2-4 |
| Supplementary Figures 1 | Pages 5 |

Supplementary Table 1 Author dispersion according to productivity level.

|  | PI≧1  (10 or more articles) | 0 < PI < 1  (2-9 articles) | PI=0  (1 articles) | Total |
| --- | --- | --- | --- | --- |
| Number of authors  (%) | 36  (0.7) | 5982  (22.4) | 20375  (76.5) | 26643  (100) |

PI: participation index.

Supplementary Table 2 Distribution of the journals in Bradford’s zones.

|  | Number of journals | Percentage (%) | Number of articles | Percentage (%) |
| --- | --- | --- | --- | --- |
| Core | 28 | 2.36% | 1984 | 33.30% |
| Zone 1 | 131 | 11.05% | 2014 | 33.80% |
| Zone 2 | 1027 | 86.59% | 1960 | 32.90% |
| Total | 1186 | 100% | 5958 | 100% |

Supplementary Table 3 The top 15 keywords related to ovarian cancer imaging.

| Rank | Keywords | Frequency | Total link strength |
| --- | --- | --- | --- |
| 1 | ovarian cancer | 1256 | 7805 |
| 2 | ultrasound | 725 | 5305 |
| 3 | diagnosis | 712 | 4853 |
| 4 | CT | 447 | 2947 |
| 5 | tumors | 442 | 2881 |
| 6 | survival | 422 | 2912 |
| 7 | women | 416 | 2745 |
| 8 | management | 416 | 2716 |
| 9 | risk | 402 | 2822 |
| 10 | chemotherapy | 401 | 2353 |
| 11 | benign | 355 | 2809 |
| 12 | positron-emission-tomography | 353 | 2433 |
| 13 | MRI | 337 | 2173 |
| 14 | expression | 325 | 1692 |
| 15 | surgery | 309 | 2132 |

**Supplementary Figure 1**


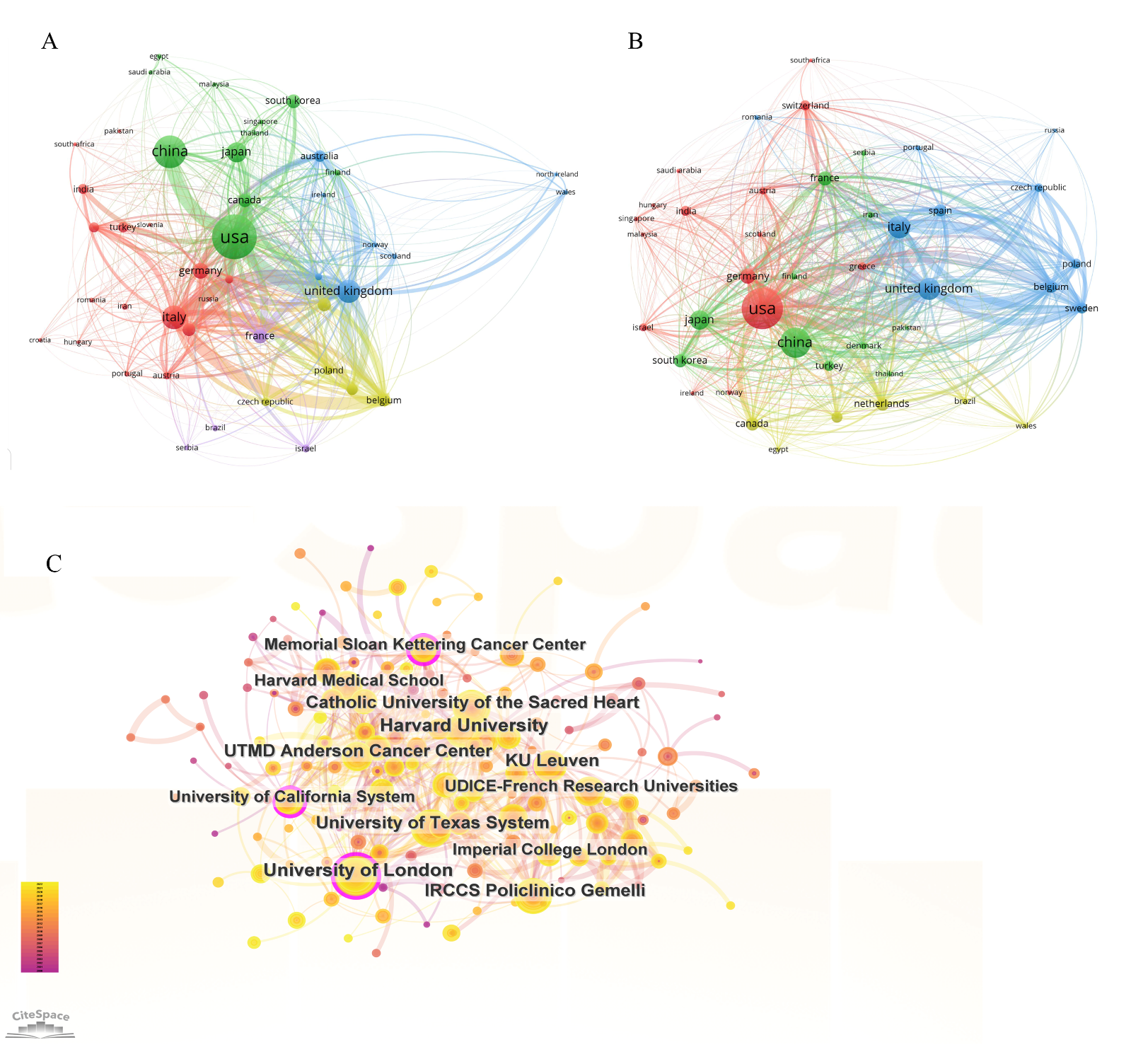


Supplementary Figure 1 Visualization of countries and institutions. The visualization network of countries/regions (A) and co-cited countries/regions (B) related to ovarian cancer imaging. The nodes with the same color belong to the same cluster, indicating a close partnership. The larger the node’s size or the thickness of the connecting line, the stronger the relative degree of co-occurrence. (C) Cooperative relationships among institutions related to ovarian cancer imaging. The nodes represent institutions. The size of nodes indicates the number of published documents, the more publications, the bigger the nodes. The connection between nodes represents the cooperation between institutions, the thickness of the lines shows the level of the combinations.
